# JNK inhibition reduces lung remodeling and pulmonary fibrotic systemic markers

**DOI:** 10.1186/s40169-016-0117-2

**Published:** 2016-09-02

**Authors:** Jos L. J. van der Velden, Ying Ye, James D. Nolin, Sidra M. Hoffman, David G. Chapman, Karolyn G. Lahue, Sarah Abdalla, Peng Chen, Yong Liu, Brydon Bennett, Nasreen Khalil, Donna Sutherland, William Smith, Gerald Horan, Mahmoud Assaf, Zebulun Horowitz, Rajesh Chopra, Randall M. Stevens, Maria Palmisano, Yvonne M. W. Janssen-Heininger, Peter H. Schafer

**Affiliations:** 1 Department of Pathology, University of Vermont, Burlington, VT USA; 2 Department of Translational Development, Celgene Corporation, 86 Morris Avenue, Summit, NJ 07901 USA; 3 Clinical Research and Development, Celgene Corporation, Warren, NJ USA; 4 Department of Inflammation Research, Celgene Corporation, San Diego, CA USA; 5 Division of Respiratory Medicine, University of British Columbia, Vancouver, BC Canada

**Keywords:** Biomarkers, CC-930, Idiopathic pulmonary fibrosis, JNK, Matrix metalloproteinase 7, Surfactant protein D, Tenascin-C

## Abstract

**Background:**

Lung remodeling and pulmonary fibrosis are serious, life-threatening conditions resulting from diseases such as chronic severe asthma and idiopathic pulmonary fibrosis (IPF). Preclinical evidence suggests that JNK enzyme function is required for key steps in the pulmonary fibrotic process. However, a selective JNK inhibitor has not been investigated in translational models of lung fibrosis with clinically relevant biomarkers, or in IPF patients.

**Methods:**

The JNK inhibitor CC-930 was evaluated in the house dust mite-induced fibrotic airway mouse model, in a phase I healthy volunteer pharmacodynamic study, and subsequently in a phase II multicenter study of mild/moderate IPF (*n* = 28), with a 4-week, placebo-controlled, double-blind, sequential ascending-dose period (50 mg QD, 100 mg QD, 100 mg BID) and a 52-week open-label treatment-extension period.

**Results:**

In the preclinical model, CC-930 attenuated collagen 1A1 gene expression, peribronchiolar collagen deposition, airway mucin MUC5B expression in club cells, and MMP-7 expression in lung, bronchoalveolar lavage fluid, and serum. In the phase I study, CC-930 reduced c-Jun phosphorylation induced by UV radiation in skin. In the phase II IPF study, there was a CC-930 dose-dependent trend in reduction of MMP-7 and SP-D plasma protein levels. The most commonly reported adverse events were increased ALT, increased AST, and upper respiratory tract infection (six subjects each, 21.4 %). A total of 13 subjects (46.4 %) experienced adverse events that led to discontinuation of study drug. Nine out of 28 subjects experienced progressive disease in this study. The mean FVC (% predicted) declined after 26–32 weeks at doses of 100 mg QD and 100 mg BID. Changes in MMP-7, SP-D, and tenascin-C significantly correlated with change in FVC (% predicted).

**Conclusions:**

These results illustrate JNK enzymatic activity involvement during pulmonary fibrosis, and support systemic biomarker use for tracking disease progression and the potential clinical benefit of this novel intervention in IPF.

*Trial registration* ClinicalTrials.gov NCT01203943

**Electronic supplementary material:**

The online version of this article (doi:10.1186/s40169-016-0117-2) contains supplementary material, which is available to authorized users.

## Background

Idiopathic pulmonary fibrosis (IPF) is a chronic, progressive, fibrosing interstitial lung disease of unknown etiology. Primarily occurring in older individuals, IPF is characterized by progressive worsening in dyspnea and pulmonary function [1]. The exact mechanism underlying IPF remains to be elucidated. According to current hypotheses, repetitive epithelial injury, aberrant wound healing, endoplasmic reticulum stress, and abnormal epithelial-mesenchymal interactions are crucial events in the fibrotic process [2–4]. Patients with IPF have a poor prognosis, with median survival of 2–4 years after diagnosis [5–8]. Recently, two drugs, pirfenidone and nintedanib, have shown efficacy in slowing disease progression in IPF, marking a significant advance in pharmacological treatment [9, 10]. However, there remains a strong unmet need for additional therapeutics that will further improve patient outcomes.

Multiple biomarkers have been proposed to be associated with IPF because they are expressed differentially in IPF patients compared with healthy individuals, are associated with survival of IPF patients, or play a known or hypothesized role in wound healing and/or the pulmonary fibrotic process [11]. Matrix metalloproteinases (MMP) degrade extracellular matrix components, and endogenous inhibitors of MMPs block their protease activity [11]. Increased MMP-1 and MMP-7 protein concentrations were observed in the plasma, bronchoalveolar lavage fluid (BALF), and lung tissue of IPF patients, with MMP-7 concentrations negatively correlated with forced vital capacity (FVC) and diffusing capacity for carbon monoxide (DL_CO_) [11]. The plasma MMP-7 level has been included, along with gender, FVC, and DL_CO_, in a predictive index of mortality in IPF patients [8]. Surfactant protein A (SP-A) and surfactant protein D (SP-D) are C-type lectins in the collectin subgroup, expressed by alveolar epithelial cells [12]. SP-A and SP-D levels in serum are elevated in patients with IPF compared with healthy subjects and/or those with other interstitial lung diseases. Moreover, increased levels of serum SP-A and SP-D have been associated with increased mortality in IPF [13, 14]. Tenascin-C, an extracellular matrix protein expressed during wound healing, has been identified at elevated levels in the fibroblast foci and in the basement membrane regions under the epithelium of honeycomb lesions in the lungs of IPF patients [15]. Myofibroblasts were found to express the highest tenascin-C levels [16]. Other proposed IPF biomarkers include Krebs von den Lungen-6 antigen (KL-6) [17], CCL-18 [18], monocyte chemotactic protein-1 (MCP-1; also known as CC chemokine ligand 2) [19], tissue inhibitor of metalloproteinase-1 (TIMP-1) [20], and plasminogen activator inhibitor-1 (PAI-1) [21].

The c-Jun N-terminal Kinase (JNK) pathway is activated by multiple cytokines and exposure to environmental stress [22]. JNK activation induces phosphorylation of c-Jun and other downstream molecules, which in turn regulate expression of genes involved in cell growth, differentiation, survival, and apoptosis. Several processes leading to fibrosis may be associated with JNK pathway activation. Multiple cell types in the lungs of IPF patients, including alveolar epithelial cells, vascular endothelial cells, alveolar macrophages, smooth muscle cells, and lymphocytes, overexpress phosphorylated JNK, with levels reflecting the degree of fibrosis [23]. The pro-fibrotic cytokine, Transforming Growth Factor β1 (TGF-β1), stimulates transformation of human lung fibroblasts into myofibroblasts that produce collagen and other extracellular matrix components, a transformation process blocked by JNK inhibition [24]. Pulmonary fibroblasts also proliferate in response to TGF-β1 through the release of fibroblast growth factor-2, which induces phosphorylation of JNK and p38 mitogen-activated protein kinases [25]. JNK1-deficient mice are protected against TGF-β1- and bleomycin-induced pro-fibrotic gene expression and pulmonary fibrosis [26].

CC-930 is a potent, selective, and orally active JNK inhibitor that competes with adenosine triphosphate in the JNK-dependent phosphorylation of c-Jun [27]. CC-930 inhibits all JNK isoforms with K_i_ (inhibitory constant) values of 5–61 nM. This compound exhibits acute anti-inflammatory effects in vivo. For example, CC-930 reduced tumor necrosis factor alpha (TNF-α) by 23 and 77 % at oral doses of 10 and 30 mg/kg, respectively, in the rat LPS-injection model [27]. To evaluate the anti-fibrotic potential of CC-930, the mouse bleomycin-induced model of lung inflammation and fibrosis was initially chosen to demonstrate chronic efficacy. CC-930 was tested prophylactically at 25, 50, 100 and 150 mg/kg prior to instillation of bleomycin, followed by 13 days of twice-daily dosing. A statistically significant inhibition of white blood cells, monocytes, and lymphocytes was observed in the bronchoalveolar lavage at all doses compared with the vehicle control, and lung fibrosis scores were reduced by 18–32 % in a dose-dependent manner [27]. CC-930 also blocked myofibroblast differentiation, collagen accumulation, and fibrosis development in a dermal fibrosis model [28]. In vitro, CC-930 suppressed pro-fibrotic gene expression by dermal fibroblasts isolated from patients with systemic sclerosis: collagen 1A1, collagen 1A2, and fibronectin mRNA levels were reduced to 71, 61 and 55 % of control by 1 μM CC-930. In the bleomycin-induced dermal fibrosis mouse model, CC-930 treatment dose-dependently reduced dermal thickening by up to 45 %, prevented the accumulation of collagen as measured by hydroxyproline content by up to 53 %, and completely inhibited the differentiation of fibroblasts into active myofibroblasts at doses of 150 mg/kg. At these doses in the TSK1 tight-skin mouse model, CC-930 reduced hypodermal thickening in a dose-dependent manner by up to 85 %, and completely prevented myofibroblast differentiation [28]. We recently reported that JNK1-deficient mice showed decreased pulmonary fibrosis without reduced inflammation in house dust mite (HDM)-induced lung remodeling in the mouse [29].

These cumulative data on CC-930 pharmacology, coupled with an appreciation for the dependency of fibrosis on the JNK pathway, as well as the remaining unmet medical needs in IPF medical treatment, led us to evaluate CC-930 in a series of nonclinical and clinical studies aimed at developing a novel therapy for IPF. Here, we show that CC-930 inhibits HDM-induced pulmonary fibrosis with minimal effects on inflammation. We further present results from two clinical studies of CC-930. The first clinical study was designed to provide proof of activity that CC-930 inhibits JNK in healthy volunteers and to select doses for evaluation in IPF patients. The second study was designed to evaluate the safety, pharmacokinetics, and pharmacodynamics of CC-930 in IPF patients. Information on disease progression was evaluated as a secondary objective. The pharmacodynamic effects of CC-930 on select blood biomarkers associated with IPF were used to assess the relationships among the CC-930 dose, exposure, efficacy, and biologic activity.

## Methods

The methodology used in the HDM-induced lung remodeling model is described in the Additional file 1.

### Study approval

All animal studies were approved by the Institutional Animal Care and Use Committee (IACUC) at the University of Vermont. Both clinical studies were conducted in accordance with the ethical principles outlined in the Declaration of Helsinki and in compliance with Good Clinical Practice and all local regulatory requirements. The study protocols were approved by the IRB or independent ethics committee at each site, and all patients provided written informed consent before enrollment.

### Experimental design

The phase I clinical study of CC-930 in healthy volunteers was a single-center, randomized, double-blind, placebo-controlled, multiple-dose, 3-way crossover study that was designed to evaluate the effect of CC-930 on JNK activity after UVB irradiation of human skin. The study was run by MDS Pharma Services (Tempe, AZ) from August to October 2009. Fifteen healthy subjects were randomized to 1 of 3 dosing sequences, with five subjects per sequence: oral placebo, CC-930 75 mg QD, or CC-930 200 mg QD for 6 days. There was a minimum of a 7-day washout between dosing periods. Compliance with medication dosing was monitored by maintaining an accurate record of all study drug administrations (including dispensing and dosing) on the test compound pages of a subject’s case report form.

The phase II clinical study of CC-930 in IPF patients was a multicenter study, open to enrollment from January 2011 to August 2012, which consisted of a screening period, 4-week blinded sequential ascending-dose phase, a 52-week open-label treatment-extension phase, and a 52-week observational follow-up phase. A total number of 19 clinical sites were activated to recruit subjects (13 sites in the United States and six sites in Canada), out of which 11 sites enrolled subjects (seven sites in the United States and four sites in Canada). The study population was restricted to subjects with an FVC >50 and <90 % of predicted, DLco >25 and <90 % of predicted, and resting saturated oxygen of >92 % on room air at sea level who have findings of usual interstitial pneumonia pattern on HRCT and/or usual interstitial pneumonia pattern on histopathology (i.e., lung biopsy), and the exclusion of known causes of interstitial lung disease. The primary endpoint of the study was to evaluate the safety (clinical adverse events (AE), laboratory, or other changes) of CC-930 in the 4-week double-blind treatment phase. Secondary endpoints included: CC-930 pharmacokinetics; evaluation of the long-term safety of CC-930 in the 52-week open-label extension; and disease progression and death rates within the total treatment and follow-up observational period. Exploratory endpoints included: changes in blood biomarkers KL-6, MMP-1, MMP-7, SP-A and SP-D, TIMP-1, CCL-18, CCL-2, PAI-1, and tenascin C; changes in FVC and DL_CO_ from baseline; and change in HRCT pulmonary fibrosis score from baseline. The double-blind, ascending-dose phase consisted of three sequential dose cohorts [CC-930 50 mg QD (cohort 1), CC-930 100 mg QD (cohort 2), and CC-930 100 mg BID (cohort 3)]. In each cohort, eligible patients were randomly allocated to receive placebo (two patients) or CC-930 (eight patients) for 4 weeks. The decision to escalate to the next dose level was made after all patients had completed or discontinued prematurely from the preceding dose level, and pharmacokinetic and safety data from the preceding dose level had been reviewed by an external data monitoring committee. Ongoing review of blinded safety data from eight patients receiving CC-930 100 mg BID (cohort 3) revealed increases in liver transaminases that may have been a potential risk to patients if they continued at that dose. This observation led to the issuing of a protocol amendment to decrease the study drug dose in Cohort 3 from 100 mg BID to 100 mg QD (3 of 8 subjects remained on study and were dose-reduced). The data from these 3 subjects were handled on an intent-to-treat basis. All patients who completed the double-blind, ascending-dose phase were eligible to enter the open-label treatment-extension phase and continue on the same dose of CC-930 for 52 weeks. Patients initially allocated to placebo were given the CC-930 dose from their original cohort assignment. The external data monitoring committee regularly reviewed safety results during this phase.

### Subjects

In the phase I clinical study, healthy male and female volunteers from any race were eligible to participate if they were aged 18–50 years and had Fitzpatrick skin type I or II. Subjects were excluded if they had any serious medical condition or history of major medical condition within 3 years of study, any prescribed systemic or topical medication within 30 days of first dose administration, or any surgical or medical conditions that could affect drug pharmacokinetics.

In the phase II IPF clinical study, patients were eligible to participate if they had confirmed IPF based on American Thoracic Society/European Respiratory Society guidelines, with an FVC of 50–90 % of predicted and a DL_CO_ of 25–90 % of predicted. Patients were excluded for resting oxygen saturation <92 % on room air at sea level or <88 % at ≥5000 feet^2^ above sea level; pulmonary arterial hypertension, emphysema, or other significant respiratory disorders; current smoker; connective tissue disorder; significant medical conditions or laboratory abnormalities or history of clinically significant medical conditions that would prevent study participation or put patients at risk; history of serious cardiac conditions within 6 months of study entry; and ECG abnormalities.

### Assessments

In the phase I clinical study, a small area of skin on the buttocks was exposed to 2 times the minimal erythema dose of UVB at 2 h after dosing on day 6. This exposure to UVB has previously been demonstrated to activate JNK and subsequently increase phosphorylation of its substrate c-Jun [31]. A skin punch biopsy was taken from the irradiated area 8 h after UVB. The skin specimen was analyzed for phospho-c-Jun. IHC images were quantified using a semi-quantitative scale from 0 to 4, in which higher scores indicated more intense immunostaining. Pharmacokinetic analyses were conducted using blood samples collected on day 1 predose and at 1, 2, 3, 6, 10, and 24 h post-dose. Safety assessments included monitoring of AEs, vital signs, 12-lead ECGs, and clinical laboratory tests.

In the phase II IPF clinical study, patients were screened within 5 weeks before the double-blind, ascending-dose phase. Study visits were scheduled at weeks 0 (baseline), 1, 2, and 4 of the double-blind, ascending-dose phase, and at weeks 5, 6, and 8, and every 4 weeks thereafter until the final treatment visit at week 56 or at the early termination visit. Subsequent observational follow-up visits were planned quarterly. Safety assessments, including AEs, vital signs, 12-lead ECGs, and clinical laboratory tests, were performed at each visit. A complete physical examination was done at screening and at the end of each treatment phase; a targeted physical examination was done at all other visits. Holter monitoring was done within 10 days of the first dose of study treatment and at weeks 1, 5, and 16. Chest radiographs were obtained at screening and at the final treatment/early termination visit.

To monitor the disease course, spirometry and DL_CO_ assessments were made at baseline, every 4 weeks until week 24, and every 8 weeks thereafter. The HRCT/fibrosis score was determined at screening and at the final treatment/early termination visit. Disease progression was defined as death from respiratory failure, progressive sustained decrease in FVC or DL_CO_, unexplained worsening of hypoxia, or acute exacerbation. Other efficacy parameters, including changes from baseline in FVC, DL_CO_, and HRCT/fibrosis score, were exploratory endpoints.

Blood was collected at baseline and at weeks 4, 24, and 56 for measurement of MMP-1, MMP-7, TIMP-1, tenascin-C, SP-A, SP-D, CCL-18, KL-6, MCP-1, and PAI-1. All ten biomarkers were assayed using validated enzyme-linked immunosorbent assays or other similar methods by Pacific Biomarkers, Inc (Seattle, WA). For the pharmacokinetics analysis, a subset of patients volunteered for intense blood sampling on day 1 and week 2; all others had sparse sampling at week 2. Sparse sampling was also done at weeks 8, 12, 16, 20, 24, 32, 40, 48, and 56. The plasma concentration of CC-930 and its major circulating and pharmacologically inactive metabolite CC-17172 were measured using a validated liquid chromatography–tandem mass spectrometry method.

### Statistical analysis

Safety parameters were evaluated in all patients who received ≥1 dose of study drug using descriptive statistics. Treatment-emergent AEs were coded to the Medical Dictionary for Regulatory Activities (version 14.0). Pharmacokinetics and pharmacodynamics were evaluated in all patients from the safety population who had ≥1 pharmacokinetic and ≥1 biomarker assessment, respectively. In the phase I study in healthy volunteers, phospho-c-Jun IHC images were scored by three independent evaluators using a subjective scoring scale of 0–4 (increases in scores reflect higher immuno-staining). Individual phospho-c-Jun scores were subtracted by the corresponding placebo to reflect the individual response to CC-930. A repeated measure logistic regression was performed on the IHC scores of total c-Jun and phospho-c-Jun using the GENMOD procedure in SAS version 9.2 (SAS Institute, Cary, NC). The model included sequence, period, and treatment as fixed effects. In the analysis, scores of 0, 1, and 2 were grouped together (low score), and scores of 3 and 4 were grouped together (high score). All 14 subjects in the pharmacodynamic population were included in the analysis. In the phase II IPF clinical study, changes from baseline in biomarker values were summarized separately for the double-blind, ascending-dose phase and the entire study using descriptive statistics.

The CC-930 plasma concentration data from the phase II IPF clinical study were combined with data from a phase I study conducted in 13 healthy subjects to build a 2-compartment population pharmacokinetics model. Population modeling and simulation were performed using NONMEM^®^ version 7.2 (Ellicott City, MD). Correlations between the model-predicted individual exposure to CC-930, as defined by the area under the plasma drug concentration-versus-time curve during the dosing interval, and the changes from baseline in MMP-7 were analyzed using a locally weighted polynomial regression method. Correlations between the changes from baseline in biomarker values versus changes from baseline in FVC percent of predicted were evaluated by Spearman’s correlation coefficient (*r*) and the associated *P* value based on the asymptotic test for H0.

## Results

### HDM-induced lung remodeling

**Fig. 1:**
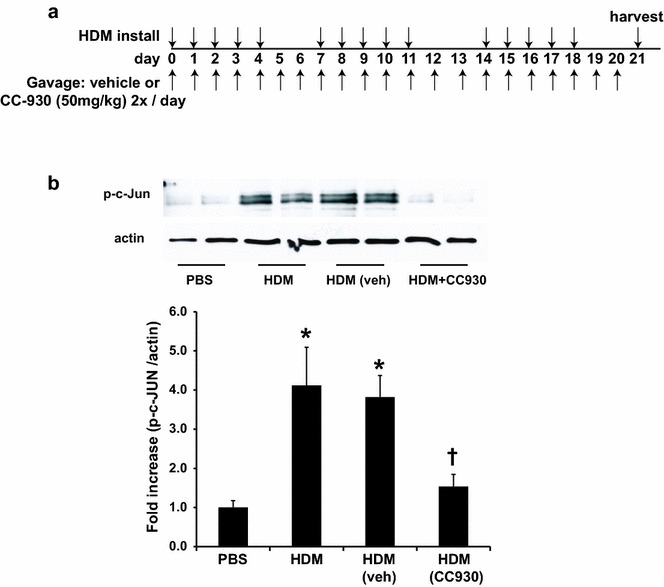
C-Jun phosphorylation in HDM-challenged mice and the impact of CC-930 or vehicle control. **a** Schematic depicting the time course of HDM exposure and CC-930 administration. For instillation, 50 µg HDM or PBS as the vehicle control was administered intranasally once for 5 days over 3 weeks at the days indicated via the *top arrows*. Vehicle control (0.5 % CMC/0.25 % Tween 80) or CC-930 (100 mg/kg) was administered twice daily on the days indicated via the *bottom arrows*. Mice were euthanized 72 h after the last challenge. **b** Lung tissue was homogenized from PBS or HDM-challenged mice for assessment of phospho-c-Jun. Equal amounts of protein (20 μg for phosho-c-jun, 5 μg for actin) were separated by sodium dodecyl sulfate–polyacrylamide gel electrophoresis (SDS–PAGE) and subjected to Western blot analysis for phospho-c-Jun and actin. Band intensity was determined and expressed as a ratio of phosphorylation to total protein (actin). Data shown represent mean ± SEM from two independent experiments (PBS: *n* = 10; HDM alone: *n* = 10; HDM + vehicle control: *n* = 11; HDM + CC-930: *n* = 11). *P < 0.05 (analysis of variance) versus PBS. ^†^ *P* < 0.05 versus the HDM group

The JNK inhibitor CC-930 had previously been tested in the mouse bleomycin-induced pulmonary fibrosis model, having shown a dose-dependent reduction in lung fibrosis scores [27]. However, in this model, MMP-7 levels were measured and found to not be induced by bleomycin instillation. Therefore, we sought a different preclinical model of lung fibrosis that might better reflect the biomarker changes seen in patients with IPF. For this reason, we explored the utility the HDM lung fibrosis model. To address the impact of CC-930 on the fibrotic airway remodeling associated with allergic airways disease, we exposed mice to the asthma-relevant allergen, HDM, over a time frame of 3 weeks, in the presence of CC-930 or vehicle control (Fig. 1a). Levels of phosphorylated c-Jun (phospho-c-Jun; reflective of JNK activation) in homogenized lung tissue were increased in mice exposed to HDM, as compared with PBS vehicle controls. As expected, the HDM-induced increase in phospho-c-Jun was attenuated in mice pretreated with CC-930 compared with vehicle controls (Fig. 1b).

### HDM-induced mucus metaplasia

**Fig. 2:**
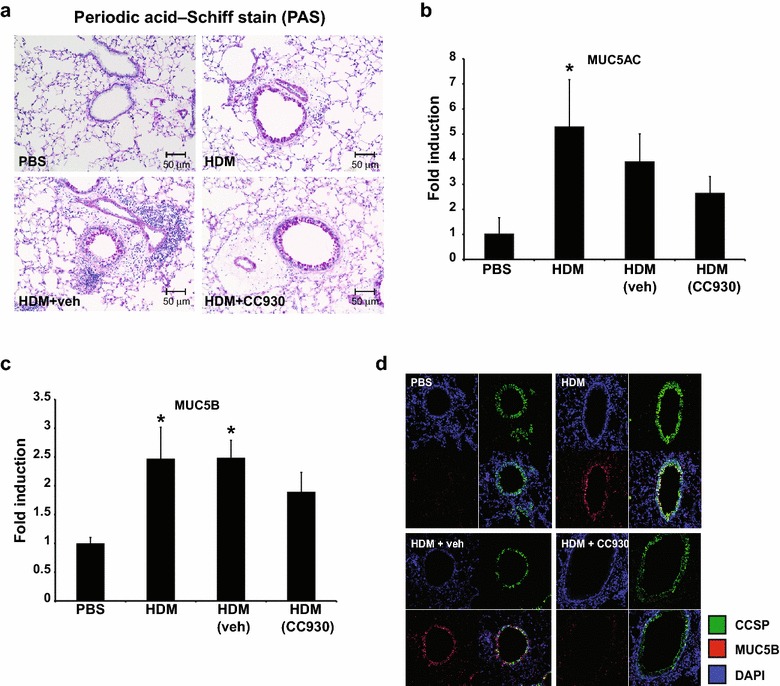
Effect of CC-930 or vehicle on HDM-induced mucus metaplasia. **a** Periodic acid-Schiff staining of airway mucus in mice exposed to PBS, HDM alone, HDM + vehicle, or HDM + CC-930 (magnification: 200×). Quantification of mRNA levels for **b** MUC5AC and **c** MUC5B in lung tissue homogenates by q-PCR after PBS or HDM exposure. **d** MUC5B immunofluorescent (*red*) and club cell secretory protein (*green*) staining of mouse lungs exposed to PBS, HDM alone, HDM + vehicle, or HDM + CC-930. Nuclei were stained using DAPI (*blue*) (magnification: 200×). Data shown represents mean ± SEM from two independent experiments (PBS: *n* = 10; HDM alone: *n* = 10; HDM + vehicle control: *n* = 11; HDM + CC-930: *n* = 11). *P < 0.05 (analysis of variance) versus PBS

We next addressed the impact of CC-930 on HDM-induced mucus metaplasia, a prominent feature of allergic airways disease. Comparable periodic acid-Schiff (PAS) reactivity was observed in CC-930-treated and vehicle-treated mice exposed to HDM (Fig. 2a). The airway mucin MUC5AC increased in CC-930-treated and vehicle-treated mice but reached statistical significance only in mice exposed to HDM (Fig. 2b). MUC5B significantly increased in both HDM vehicle control groups, and again did not reach significance in the CC-930-treated mice (Fig. 2c). Assessment of MUC5B by immunofluorescence showed strong increases in MUC5B reactivity in club cell secretory protein positive cells after HDM exposure, which was attenuated after administration of CC-930 (Fig. 2d). Overall, these findings demonstrate that JNK did not appear to play a dominant role in mucus metaplasia despite the slight attenuations of MUC5B that were observed in CC-930-treated mice.

### HDM-induced airway remodeling

**Fig. 3:**
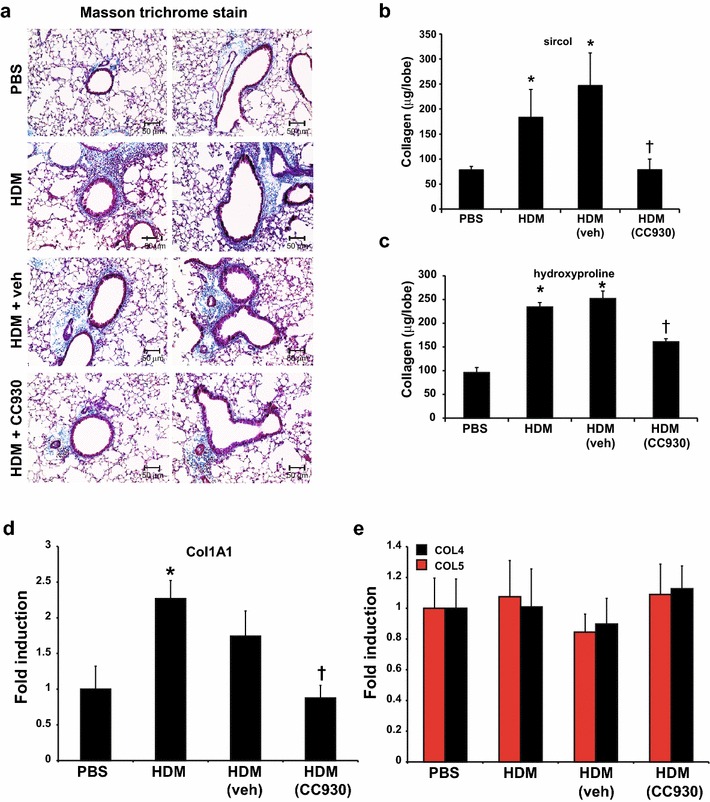
Impact of CC-930 or vehicle on HDM-induced fibrotic airway remodeling. **a** Histopathological analysis of Masson’s trichrome-stained airway sections in mice exposed to PBS, HDM alone, HDM + vehicle, or HDM + CC-930 (magnification: 200×). Assessment of total collagen content by **b** sircol and **c** hydroxyproline assay in the upper right lobe of mice after PBS, HDM alone, HDM + vehicle, or HDM + CC-930 exposure. Assessment of mRNA abundance of **d** collagen 1A1 and **e** collagen 4, collagen 5, and mRNA abundance was normalized to β-actin. Results are expressed as fold change compared with PBS-exposed mice and reflect mean ± SEM from two independent experiments (PBS: *n* = 10; HDM alone: *n* = 10; HDM + vehicle control: *n* = 11; HDM + CC-930: *n* = 11). *P < 0.05 (analysis of variance) versus PBS. ^†^ *P* < 0.05 compared with the HDM group

The effect of CC-930 on HDM-induced fibrotic airway remodeling was analyzed histopathologically using the Masson’s trichrome collagen stain. In response to HDM, collagen content was significantly enhanced around the bronchioles, consistent with earlier observations [29]. Remarkably, in mice receiving CC-930, the HDM-induced peribronchiolar deposition of collagen was significantly attenuated, while vehicle-treated mice exposed to HDM showed increases in peribronchiolar collagen deposition similar to those observed in HDM-exposed mice (Fig. 3a). Quantitative assessment of soluble and total lung collagen content demonstrated decreases in CC-930-treated mice compared with vehicle controls exposed to HDM (Fig. 3b, c). HDM-mediated increases in collagen 1A1 mRNA also were reduced in CC-930-treated mice compared with HDM vehicle controls (Fig. 3d). In comparison, α-smooth muscle actin (α-SMA), E-cadherin, and SP-D (see Additional file 1: Figure S1), as well as collagen 4 and collagen 5, were not significantly affected in mice exposed to HDM (Fig. 3e). TGF-β1 is a major pro-fibrogenic growth factor that has been implicated in lung fibrosis, and cooperation between JNK and TGF-β1 signaling has been observed [30]. HDM led to marked increases of TGF-β1 in bronchoalveolar lavage, which was not affected by CC-930 or vehicle, suggesting that TGF-β1 is activated proximally to or independent of JNK (see Additional file 1: Figure S2a). Overall, these findings demonstrate that CC-930 had a protective effect on lung collagen deposition in mice exposed to HDM.

### HDM-induced airway inflammation and airway hyperresponsiveness

Major features of HDM-induced disease are airway inflammation and hyperresponsiveness to methacholine. HDM-induced airway inflammation and hyperresponsiveness were not significantly affected by CC-930 (Additional file 1: Figure S3). These findings are consistent with prior observations in JNK1-/- mice exposed to HDM, which showed patterns of airway inflammation and hyperresponsiveness comparable to wild-type mice [29].

### HDM-induced MMP-7 protein and gene expression

**Fig. 4:**
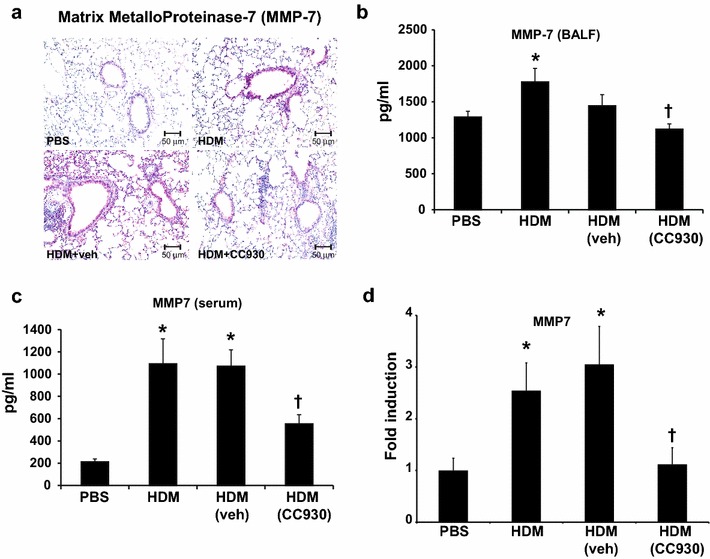
HDM-induced MMP-7 protein and gene expression is attenuated by CC-930. **a** MMP-7 immunohistochemistry staining of mouse lungs exposed to PBS, HDM alone, HDM + vehicle, or HDM + CC-930 (magnification: 200×). **b** Evaluation of total MMP-7 protein in BALF and **c** serum measured by the enzyme-linked immunosorbent assay and **d** mRNA expression of MMP-7 in lung homogenates measured by q-PCR mice after PBS or HDM exposure. Data shown represents mean ± SEM from two independent experiments (PBS: *n* = 10; HDM alone: *n* = 10; HDM + vehicle control: *n* = 11; HDM + CC-390: *n* = 11). *P < 0.05 (analysis of variance) versus PBS. ^†^ *P* < 0.05 versus the HDM + vehicle group

MMP-7 has been identified as a biomarker for IPF [11]. An IHC evaluation of MMP-7 revealed robust increases in reactivity in lung tissues from HDM-treated mice compared with PBS-treated mice, with notable reactivity occurring in the bronchiolar epithelium (Fig. 4a). Similarly, after HDM exposure, increases in MMP-7 were observed in BALF (Fig. 4b) and serum (Fig. 4c). mRNA expression of MMP-7 was also increased in homogenized lung tissue from mice exposed to HDM, as compared with PBS vehicle controls (Fig. 4d). Administration of CC-930, but not vehicle control, led to attenuation in HDM-mediated increases in MMP-7. These findings suggest a role for JNK in regulating MMP-7 expression in settings of fibrotic airway remodeling.

### Phase I pharmacokinetic, pharmacodynamic, and safety study of CC-930 in healthy volunteers

Fifteen subjects were enrolled, and 14 (93.3 %) completed each of the three 6-day study periods (treatment with placebo, CC-930 75 mg QD, and CC-930 200 mg QD). The mean age of the study cohort was 31 years (range: 20–49 years); 100 % were white and 100 % were male. CC-930 administered for 6 days at doses of 75 mg QD and 200 mg QD was well tolerated in the healthy subjects. No subject discontinued because of an adverse event (AE), and no serious AEs were reported.

The mean CC-930 plasma concentration was 0.81 µM at the 75 mg QD dose and 1.89 µM at the 200 mg QD dose. The ability of CC-930 to inhibit JNK was determined by measuring phospho-c-Jun by IHC in skin punch biopsy specimens at 8 h after UVB exposure (Additional file 1: Figure S4). CC-930 partially or nearly completely inhibited the phospho-c-Jun elevation in 8 of 14 (57.1 %) subjects receiving 75 mg QD and in 11 of 14 (78.6 %) subjects receiving 200 mg QD. The median IHC score for phospho-c-Jun on a semi-quantitative 0–4 scale was 4 with placebo, 3 with CC-930 75 mg QD, and 1 with CC-930 200 mg QD. The odds ratio of achieving a low IHC score (0, 1, or 2) was 11.0 with CC-930 75 mg QD (*P* = 0.016) and 41.9 with CC-930 200 mg QD (*P* = 0.005).

### Phase II efficacy, safety, and pharmacodynamic study of CC-930 in patients with IPF

The study design is shown in Additional file 1: Figure S5. In total, 28 IPF patients were randomized to study treatment. Of these, 26 (92.9 %) patients completed the 4-week, placebo-controlled, double-blind, ascending-dose phase and entered the 52-week open-label treatment-extension phase (Additional file 1: Figure S6). The study cohort had a mean age of 67.1 years; most patients were white (92.9 %) and male (71.4 %) (Table 1). Patient demographics were generally well balanced across treatment groups; however, patients allocated to CC-930 100 mg QD were younger and more obese than those in the other groups. Baseline disease characteristics were generally well balanced across treatment groups. Diagnosis of IPF was conducted via high-resolution CT (HRCT) for a majority of subjects (60.7 %); however, a greater majority of 100 mg QD subjects were diagnosed with IPF via histopathology (75.0 %) compared with placebo (0.0 %), 50 mg QD (12.5 %), and 100 mg BID (0.0 %) subjects. The mean FVC was 67.1 % of predicted and the mean DL_CO_ was 41.5 % of predicted. Six patients entered the study receiving supplemental oxygen. Prior medication use was generally similar across the treatment groups; however, the proportion of patients with prior respiratory system therapies was lower for the CC-930 100 mg BID group (29 %) compared with the placebo, CC-930 50 mg QD, and CC-930 100 mg QD dose groups (60, 63 and 88 %, respectively).

**Table 1:** Baseline patient demographic and clinical characteristics in the idiopathic pulmonary fibrosis study

	Placebo *n* = 5	CC-930			All patients *N* = 28
		50 mg QD *n* = 8	100 mg QD *n* = 8	100 mg BID *n* = 7	
Age, mean (±SD), years	67.2 (±3.90)	70.0 (± 6.82)	62.0 (±7.98)	69.7 (±7.02)	67.1 (±7.33)
Males, n (%)	3 (60.0)	8 (100.0)	5 (62.5)	4 (57.1)	20 (71.4)
Race, n (%)					
Black	0 (0.0)	0 (0.0)	1 (12.5)	0 (0.0)	1 (3.6)
Native Hawaiian/Pacific Islanders	0 (0.0)	1 (12.5)	0 (0.0)	0 (0.0)	1 (3.6)
White	5 (100.0)	7 (87.5)	7 (87.5)	7 (100.0)	26 (92.9)
Weight, mean (±SD), kg	85.6 (±8.51)	96.8 (±16.58)	93.4 (±15.02)	76.0 (±16.49)	88.6 (±16.46)
Body mass index, mean (±SD), kg/m^2^	28.8 (±2.70)	29.7 (±4.23)	31.1 (±2.62)	27.0 (±2.53)	29.3 (±3.37)
FVC percent of predicted, mean (±SD)	62.3 (±12.10)	68.2 (±6.84)	66.2 (±11.56)	70.2 (±5.58)	67.1 (±9.10)
DL_CO_ percent of predicted, mean (±SD)	38.6 (±4.44)	39.3 (±7.05)	45.2 (±15.29)	41.9 (±7.28)	41.5 (±9.76)
SpO_2_ mean (±SD), %	96.6 (±1.95)	95.1 (±1.81)	96.0 (±1.31)	94.7 (±1.70)	95.5 (±1.73)
Supplemental oxygen use, n (%)	1 (20.0)	0 (0.0)	3 (37.5)	2 (28.6)	6 (21.4)

*FVC* forced vital capacity; *DL*_*CO*_ diffusing capacity for carbon monoxide; *SpO*_2_ saturation of peripheral oxygen

Patients were exposed to CC-930 and placebo for a mean of 3.8 and 4.0 weeks, respectively, during the double-blind, ascending-dose phase. Total mean exposure to CC-930 during the entire treatment period, including that of patients who switched from placebo on entry into the open-label treatment-extension phase, was 25.9 weeks. Mean exposure decreased in a dose-dependent manner: 38.8 weeks with CC-930 50 mg QD, 22.5 weeks with CC-930 100 mg QD, and 7.8 weeks with CC-930 100 mg BID. Three patients receiving CC-930 100 mg BID were switched to CC-930 100 mg QD based on an ongoing blinded safety review and received the lower dose for a mean of 21.2 additional weeks.

### Safety

The frequency of AEs during the double-blind, ascending-dose phase appeared to increase in a dose-related manner with CC-930 (12.5 % with 50 mg QD, 62.5 % with 100 mg QD, and 71.4 % with 100 mg BID) (Table 2). The only AEs experienced by ≥2 patients were dizziness (14.3 %), upper respiratory tract infection (7.1 %), headache (7.1 %), and nausea (7.1 %); in each case, the AEs occurred in patients receiving CC-930 at a dose of 100 mg QD or 100 mg BID. When the double-blind and open-label treatment-extension phases were combined, the most frequently reported AEs were increased alanine aminotransferase (ALT), increased aspartate aminotransferase (AST), and upper respiratory tract infection (each 21.4 %), as well as bronchitis, nasopharyngitis, dizziness, and increased lactate dehydrogenase (each 14.3 %). The increases in ALT, AST, and lactose dehydrogenase mostly occurred in patients receiving a dose of 100 mg BID. No deaths were reported during the CC-930 treatment period. Two patients died during the observational follow-up period; the causes of death were respiratory failure and secondary due to IPF. Both patients had received their last dose of CC-930 100 mg QD at least 50 days before their death. Three patients had serious AEs, including 1 during the double-blind, ascending-dose phase (*Clostridium difficile* colitis), and 2 during the open-label treatment-extension phase (pneumonia and increased ALT/AST).

**Table 2:** Overview of AEs in the idiopathic pulmonary fibrosis study

Double-blind treatment period, patients, *n* (%)	Placebo^a^ *n* = 5	CC-930			
		50 mg QD *n* = 8	100 mg QD *n* = 8	100 mg BID *n* = 7	Total *N* = 28
≥1 AE	3 (60.0)	1 (12.5)	5 (62.5)	5 (71.4)	14 (50.0)
≥1 severe AE	0 (0.0)	0 (0.0)	0 (0.0)	0 (0.0)	0 (0.0)
≥1 serious AE	0 (0.0)	0 (0.0)	0 (0.0)	1 (14.3)	1 (3.6)
AE leading to drug withdrawal	0 (0.0)	0 (0.0)	1 (12.5)^b^	1 (14.3)	2 (7.1)
Death	0 (0.0)	0 (0.0)	0 (0.0)	0 (0.0)	0 (0.0)
AE in ≥2 patients (in any treatment group)					
Dizziness	0 (0.0)	0 (0.0)	2 (25.0)	2 (28.6)	4 (14.3)
Upper respiratory tract infection	0 (0.0)	0 (0.0)	1 (12.5)	1 (14.3)	2 (7.1)
Headache	0 (0.0)	0 (0.0)	2 (25.0)	0 (0.0)	2 (7.1)
Nausea	0 (0.0)	0 (0.0)	2 (25.0)	0 (0.0)	2 (7.1)

CC-930 exposure period, patients, *n* (%)		CC-930			Total *N* = 28
		50 mg QD *n* = 10	100 mg QD *n* = 10	100 mg BID *n* = 8	
≥1 AE		7 (70.0)	9 (90.0)	8 (100.0)	24 (85.7)
≥1 severe AE		0 (0.0)	2 (20.0)	1 (12.5)	3 (10.7)
≥1 serious AE		0 (0.0)	1 (10.0)	2 (25.0)	3 (10.7)
AE leading to drug withdrawal		2 (20.0)	5 (50.0)	6 (75.0)	13 (46.4)
Death		0 (0.0)	0 (0.0)	0 (0.0)	0 (0.0)
AE in ≥2 patients (in any treatment group)					
Increased alanine aminotransferase		0 (0.0)	1 (10.0)	5 (62.5)	6 (21.4)
Increased aspartate aminotransferase		0 (0.0)	1 (10.0)	5 (62.5)	6 (21.4)
Upper respiratory tract infection		2 (20.0)	2 (20.0)	2 (25.0)	6 (21.4)
Increased blood lactate dehydrogenase		0 (0.0)	0 (0.0)	4 (50.0)	4 (14.3)
Bronchitis		2 (20.0)	2 (20.0)	0 (0.0)	4 (14.3)
Nasopharyngitis		1 (10.0)	2 (20.0)	1 (12.5)	4 (14.3)
Dizziness		0 (0.0)	2 (20.0)	2 (25.0)	4 (14.3)
Headache		1 (10.0)	2 (20.0)	0 (0.0)	3 (10.7)
Sinusitis		1 (10.0)	1 (10.0)	1 (12.5)	3 (10.7)
Diarrhea		0 (0.0)	3 (30.0)	0 (0.0)	3 (10.7)
Nausea		0 (0.0)	2 (20.0)	0 (0.0)	2 (7.1)
Viral gastroenteritis		0 (0.0)	1 (10.0)	1 (12.5)	2 (7.1)
Increased C-reactive protein		0 (0.0)	2 (20.0)	0 (0.0)	2 (7.1)
Muscle spasms		0 (0.0)	1 (10.0)	1 (12.5)	2 (7.1)
Musculoskeletal chest pain		0 (0.0)	2 (20.0)	0 (0.0)	2 (7.1)
Rash		1 (10.0)	0 (0.0)	1 (12.5)	2 (7.1)
Cough		1 (10.0)	1 (10.0)	0 (0.0)	2 (7.1)
Productive cough		1 (10.0)	0 (0.0)	1 (12.5)	2 (7.1)

*AE* adverse event; *ALT* alanine aminotransferase; *AST* increased aspartate aminotransferase

^a^Includes placebo patients from groups receiving CC-930 50 mg QD, 100 mg QD, and 100 mg BID

^b^Patient discontinued after completing the double-blind treatment phase but before enrolling in the open-label treatment phase

Overall, 13 patients discontinued CC-930 because of AEs, with the frequency increasing in a dose-related manner (20 % with 50 mg QD, 50 % with 100 mg QD, and 75 % with 100 mg BID). The only AEs resulting in discontinuation of >1 patient were increased lactate dehydrogenase (*n* = 4), increased ALT (*n* = 3), and increased AST (*n* = 3), all of which occurred in patients receiving CC-930 100 mg BID. The six patients who discontinued because of increased ALT or AST were the only patients with ALT or AST values >3 times the upper limit of normal. None of these cases was accompanied by concurrent alkaline phosphatase or bilirubin values >2 times the upper limit of normal, and none met the criteria for Hy’s law. All hepatic transaminase elevations resolved within 2–4 weeks after discontinuation of CC-930. Three patients discontinued because of cardiac-related AEs; it is not known if these were related to the age and health of the patients or occurred as a consequence of CC-930. Because the benefit/risk profile of CC-930 did not support continuation, this study was terminated early during the extension phase.

### Pharmacokinetics

CC-930 was rapidly absorbed following oral administration, with mean peak plasma drug concentrations achieved by approximately 1 h (Additional file 1: Figure S7). Pharmacokinetic parameters were determined for four patients randomized to CC-930 100 mg QD who participated in the intensive pharmacokinetic substudy. None of the patients allocated to CC-930 50 mg QD or CC-930 100 mg BID volunteered for this substudy. After the first dose, the mean peak plasma concentration of CC-930 was 674 ng/mL and the mean exposure until 8 h post-dose (AUC_0–8_) was 3339 ng·h/mL. These values increased to 1040 ng/mL and 5912 ng·h/mL, respectively, when measured after daily dosing for 2 weeks. The accumulation ratio for CC-930 was approximately 1.8-fold, consistent with a terminal half-life of 25–31 h, as previously measured in healthy volunteers (data not shown). Because samples were taken only until 8 h postdose in this IPF study, the PK parameters of terminal phase half-life, apparent total clearance, and apparent volume of distribution cannot be accurately determined in this patient population and are therefore not reported.

### Pharmacodynamics

**Fig. 5:**
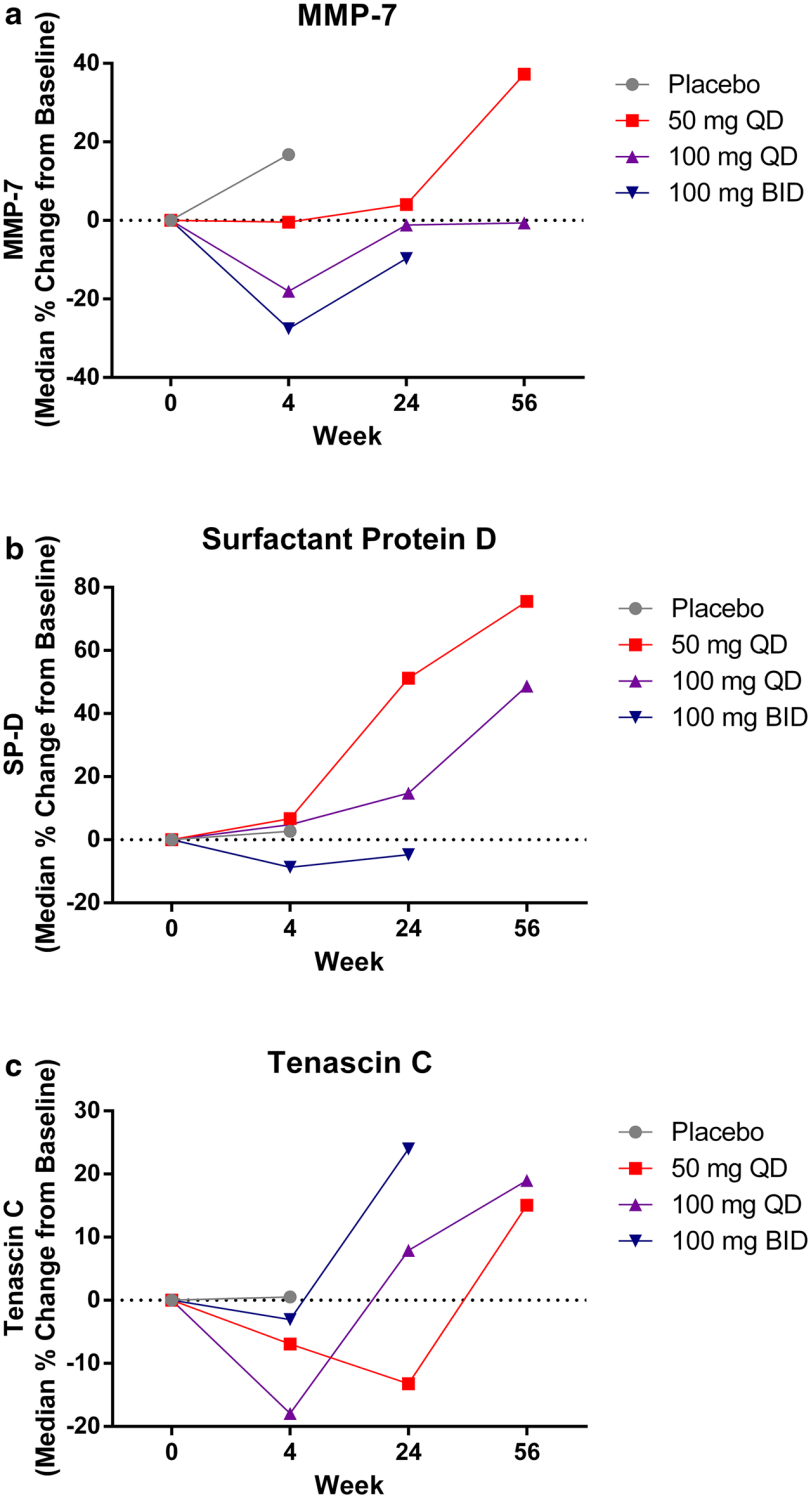
Median change from baseline in **a** plasma MMP-7, **b** surfactant protein D, and **c** tenascin-C in all treatment groups throughout the 56-week study

**Fig. 6:**
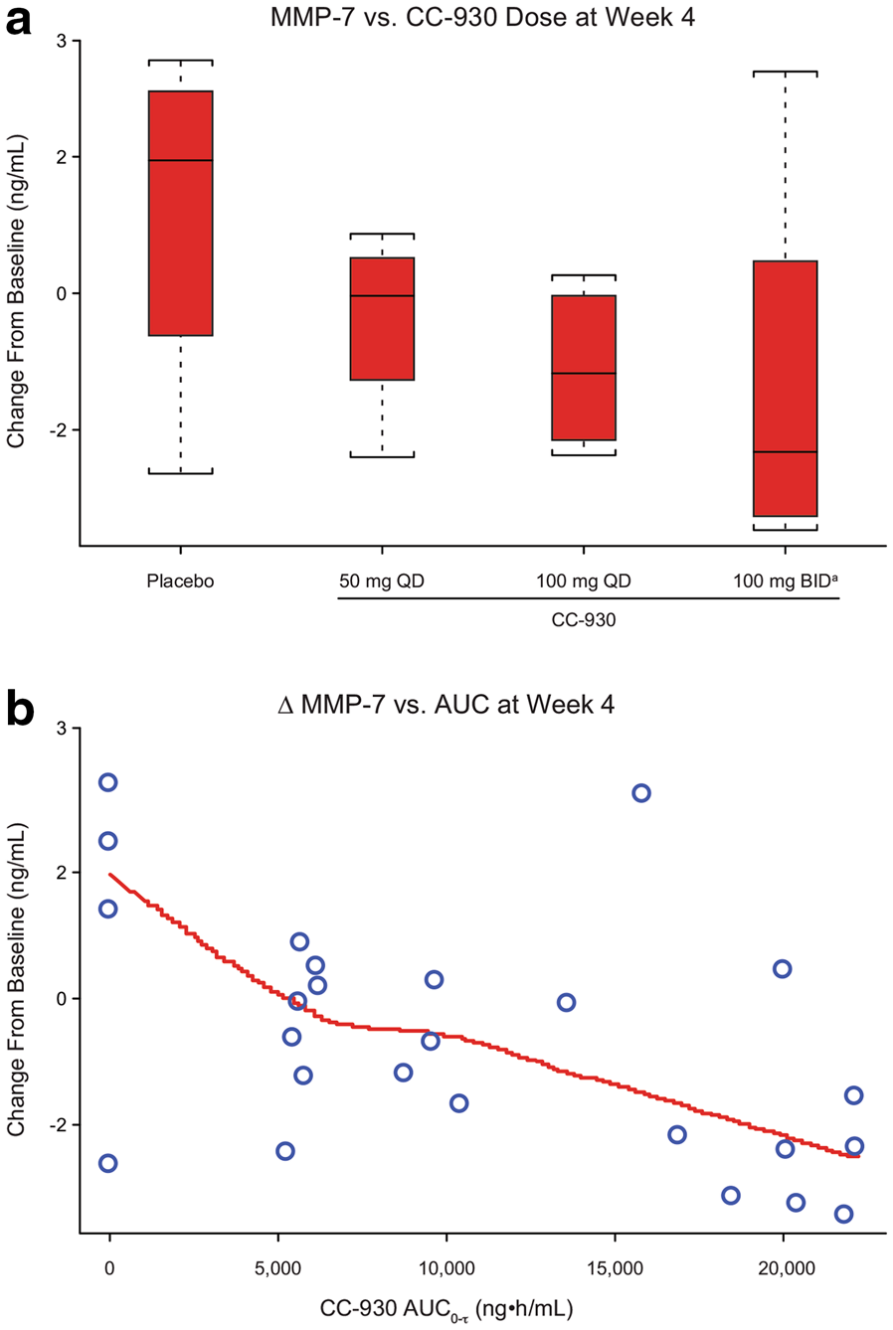
Median change from baseline in **a** plasma MMP-7 concentration at week 4 versus CC-930 dose and **b** MMP-7 versus CC-930 exposure (AUC_0−τ_) predicted from the population pharmacokinetic model. **a** The dose was reduced from 100 mg BID to 100 mg QD within the first 4 weeks for one patient due to a protocol amendment

CC-930 reduced median plasma MMP-7 concentrations in a dose-related manner during the double-blind, ascending-dose phase; the same trend continued during the open-label treatment-extension phase (Fig. 5a). The changes in plasma MMP-7 concentrations from baseline to week 4 were correlated with the CC-930 dose (Fig. 6a) and with exposure predicted from the population pharmacokinetic model (Fig. 6b). The percent changes from baseline in SP-D concentrations trended lower with increasing CC-930 dose (Fig. 5b). CC-930 at doses of 50 mg QD and 100 mg QD lowered median tenascin-C levels during the double-blind, ascending-dose phase, but little change was observed in patients receiving CC-930 100 mg BID. During the open-label treatment-extension phase, tenascin-C concentrations returned toward baseline over time and then surpassed baseline levels by the final assessment (Fig. 5c). No trends were observed for the other 7 biomarkers assayed (data not shown).

### Efficacy

**Fig. 7:**
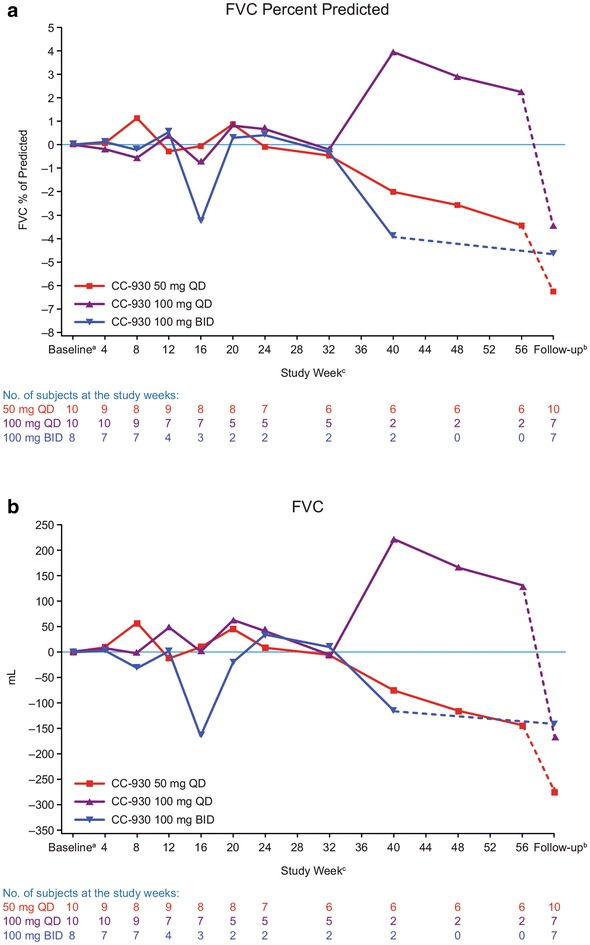
Mean change from baseline in FVC over time in idiopathic pulmonary fibrosis patients treated with CC-930. **a** FVC percent predicted and **b** FVC in milliliters. **a** Defined as the last measurement on or before the day of the first dose of study drug. **b** 4 weeks post-treatment visit. The follow-up visit appears as a separate study week in the figure, with a *dotted line* connecting the last visit where subjects’ received drug to the follow-up visit. **c** Relative to the day of the first dose of study drug

**Fig. 8:**
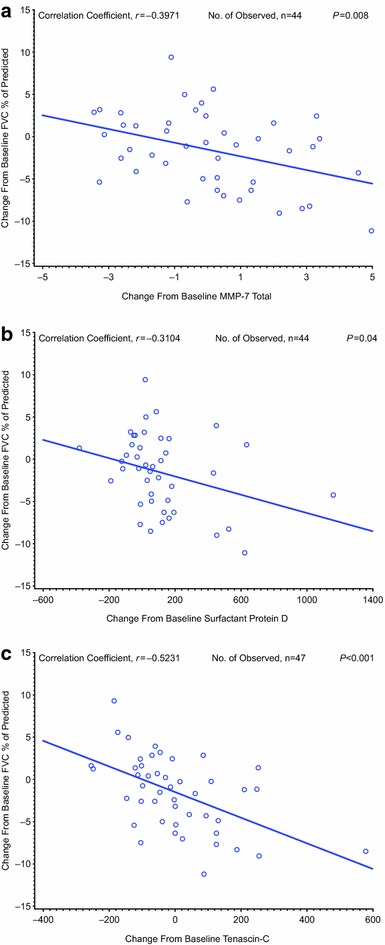
Correlation of **a** plasma MMP-7, **b** surfactant protein D, and **c** tenascin-C concentration with changes in FVC percent of predicted

Forced vital capacity remained largely unchanged in this study through week 32 (Fig. 7). Notably, there was a pronounced decline in FVC after drug withdrawal. Disease progression was reported for nine (32.1 %) patients. Three (30.0 %) patients who received CC-930 50 mg QD and five (50.0 %) patients who received CC-930 100 mg QD showed disease progression during the 4-week observational follow-up period. One (12.5 %) patient who received CC-930 100 mg BID showed disease progression on day 50 during the open-label treatment-extension phase. When all visits were considered for all patients, the changes in FVC percent of predicted were correlated with changes in plasma concentrations of MMP-7 (*r* = −0.397; *P* = 0.008), SP-D (*r* = −0.310; *P* = 0.04), and tenascin-C (*r* = −0.523; *P* < 0.001) (Fig. 8).

The HRCT/fibrosis score at the patients’ last on-treatment visit was compared with their baseline score. The median duration between the baseline and last HRCT/fibrosis score was 58.3 weeks for patients receiving CC-930 50 mg QD, 25.0 weeks for patients receiving CC-930 100 mg QD, and 16.3 weeks for patients receiving CC-930 100 mg BID. The HRCT/fibrosis score suggested disease stabilization instead of progression in 1 of 9 (11.1 %) patients receiving CC-930 50 mg QD, 6 of 10 (60.0 %) receiving CC-930 100 mg QD, and 3 of 7 (42.9 %) receiving CC-930 100 mg BID. HRCT data were missing for the other 2 patients. Improvements in reticular abnormality and honeycombing were not observed.

During the double-blind treatment phase, the classes of concomitant medications used in at least 50 % of overall subjects were cardiovascular system (75.0 %), alimentary tract and metabolism (71.4 %), and respiratory system (57.1 %). The frequency of subjects with concomitant use of respiratory system therapies was lower for the 100 mg BID group (42.9 %) compared with the placebo, 50 mg QD, and 100 mg QD dose groups (60.0, 50.0 and 75.0 %, respectively). During the open-label treatment phase, the classes of concomitant medications used in at least 50 % of subjects were cardiovascular system (84.6 %), alimentary tract and metabolism (73.1 %), respiratory system (73.1 %), nervous system (61.5 %), anti-infectives for systemic use (57.7 %), blood and blood-forming organs (53.8 %), and musculoskeletal system (53.8 %). As in the double-blind treatment phase, the frequency of subjects with concomitant use of respiratory system therapies was lower for cohort 3 (42.9 %) compared with the cohorts 1 and 2 (88.9 and 80.0 %, respectively).

## Discussion

JNK1 recently has been shown to play an important role in the pathogenesis of bleomycin and TGF-β1-induced lung fibrosis [26] and in HDM-induced fibrotic airway remodeling, while not affecting inflammatory cell recruitment, airway hyperresponsiveness, or mucus metaplasia [29]. In the present study, we demonstrated that CC-930, the pharmacological inhibitor of JNK, inhibited HDM-mediated increases in phospho-c-Jun in lung tissue. CC-930 did not affect HDM-induced inflammation, airway hyperresponsiveness, or mucus metaplasia, consistent with earlier observations using JNK1-/- mice [29]. However, our data show that CC-930 significantly reduced MMP-7 protein and mRNA expression, and attenuated peribronchiolar fibrotic remodeling and collagen deposition. These preclinical results support the potential of CC-930 as a viable candidate for treating fibrotic airways disease.

Results from the healthy volunteer phase I study demonstrate that CC-930 inhibits JNK in human subjects in a dose-related manner. JNK inhibition was determined by IHC of phospho-c-Jun in UVB-exposed skin, a model that depends on JNK activation and subsequent phosphorylation of its substrate c-Jun [31]. At the time of skin biopsy, mean CC-930 plasma concentration was 0.81 µM with the 75 mg QD dose and 1.89 µM with the 200 QD dose. These concentrations were comparable to IC_50_ values determined in cell-based studies. CC-930 inhibited UVB-induced phospho-c-Jun in cultured normal human epidermal keratinocytes with IC_50_ values between 0.1 and 3.0 μM, and it reduced growth factor-induced c-Jun phosphorylation and basal collagen protein expression in human fibroblasts from scleroderma patients at a concentration of 1 μM [28]. Results from this proof-of-concept study were therefore consistent with the cell culture studies and provided the basis for the doses selected in the phase II study.

The IPF phase II study enrolled a well-defined population of IPF patients with mild to moderate pulmonary function impairment. The primary objective was to evaluate the pharmacokinetics, pharmacodynamics, biologic activity, and safety of CC-930. Pharmacokinetic data indicated that CC-930 is rapidly absorbed following oral administration, with pharmacokinetic parameters similar to those observed previously in phase 1 participants. The change in FVC was an exploratory endpoint, and was included in the definition of disease progression used herein. Deterioration in FVC ≥10 % from baseline over 6 months has been shown to predict mortality in IPF patients [32]. The mean FVC percent of predicted remained relatively stable at least through week 32. Moreover, only 9 (32.1 %) patients experienced disease progression; for eight of these patients, it was identified during the observational follow-up period. These findings suggest that CC-930 may stabilize disease as long as patients remain on treatment. However, no definitive conclusion can be drawn on whether these data reflect the natural history of disease or are an actual benefit of CC-930 treatment. The apparently greater benefit of the higher doses of CC-930 may have reflected an imbalance in risk of progression despite the randomization scheme. Patients receiving CC-930 100 mg BID had a higher baseline FVC percent of predicted than the other patients and also had a lower history of smoking and required fewer respiratory medications at baseline.

In the pharmacodynamic assessment, CC-930 reduced plasma MMP-7 and SP-D concentrations in a dose-dependent manner. Notably, the reduction in MMP-7 correlated with the population pharmacokinetic model-predicted exposure to CC-930, and the changes in plasma MMP-7 and SP-D significantly correlated with changes in pulmonary function (FVC percent of predicted) when data for all patients and from all visits were considered. As noted, elevated MMP-7 levels have been identified in IPF patients and are negatively correlated with FVC and DL_CO_ [11]. Moreover, higher plasma MMP-7 levels have been associated with mortality in IPF patients [8]. CC-930 also reduced plasma tenascin-C concentrations, at least at the two lower doses, and the changes in plasma tenascin-C were significantly correlated with FVC percent of predicted. However, at the highest dose, tenascin-C levels were only marginally reduced from baseline. Tenascin-C, an extracellular matrix glycoprotein expressed during wound healing, is a marker of both pulmonary fibrosis and hepatitis [33]. At the lower doses, the reduction in tenascin-C levels may have been driven by a beneficial drug effect in the lungs. At the highest dose, however, the drug’s effect on the liver may have increased plasma tenascin-C levels and countered any reduction achieved from a favorable lung effect.

The JNK pathway plays an important role in the regulation of gene expression of MMP-7, SP-D, and tenascin-C. For example, mesothelin enhances the invasiveness of ovarian cancer cells by inducing MMP-7 through activation of mitogen-activated protein kinase/extracellular signal-regulated kinase and JNK pathways [34]; the human T cell leukemia virus type 1 Tax protein transactivates the MMP-7 gene via JunD/AP-1 signaling that is dependent on activation of JNK1/2 and extracellular signal-regulated kinase 1/2 [35]; and (-)-epigallocatechin-3-gallate stimulates pro-MMP-7 production via activation of the JNK1/2 pathway in HT-29 human colorectal cancer cells [36]. Regulation of SP-D gene activity depends on interactions among relatively ubiquitous transcription factors, with the conserved AP-1 site required for maximal promoter activity [37]. Tenascin-C gene expression in rat mesangial cells was down-regulated by CC-930 10 μM (data on file, Celgene Corporation). Therefore, the observed downward trends in MMP-7, SP-D, and tenascin-C expression by CC-930 treatment in the phase II clinical study are all biologically plausible. The observed correlations between the change in each of these proteins and the change in FVC percent of predicted suggest that JNK inhibition produces positive trends in pharmacodynamic markers that may be indicative of positive trends in pulmonary function in IPF patients. Whether these findings are reproducible, and whether a true positive effect in pulmonary function can be realized in an IPF population, will require further clinical studies with larger sample sizes and sufficient study duration.

The initial 4-week, double-blind, ascending-dose phase did not reveal any meaningful difference in safety and tolerability between CC-930 and placebo. Although the frequency of AEs was higher in patients receiving CC-930 100 mg QD or 100 mg BID than in patients receiving CC-930 50 mg QD, it was still largely similar to the patients receiving placebo. All but two patients completed the double-blind, ascending-dose phase and entered the open-label treatment extension. Elevated hepatic transaminases were observed in patients receiving the highest doses of CC-930, mostly occurring after 8–12 weeks of treatment. No proximate cause or risk factor was identified in any of these patients except for CC-930 treatment. Although these safety data indicate a dose-dependent, late-onset transaminitis that may be associated with liver injury, none of the cases met the criteria for Hy’s law, and each case resolved within 2–4 weeks of discontinuing CC-930. Because of these observations, the study protocol was amended to reduce the dose of CC-930 from 100 mg BID to 100 mg QD for the three patients who remained in the study on treatment. The effect of CC-930 was subsequently investigated in human and rat primary hepatocytes in vitro. No cytotoxicity was observed at clinically relevant concentrations of CC-930, and no specific toxicity related to the etiology of the transaminase elevations was identified (data not shown). In the literature, there are conflicting reports regarding the role of JNK in protection from liver damage. JNK1 knockout mice are resistant to high fat-diet induced excessive weight gain, insulin resistance, and steatohepatitis. While JNK2 knockout mice develop obesity and insulin resistance similarly to normal mice, they exhibit increased ALT enzyme levels and liver cell apoptosis compared to normal mice high fat diet-induced [38]. In mice fed a choline-deficient L-amino acid-defined (CDAA) diet, JNK1 knockout mice had less hepatic inflammation and fibrosis than normal mice, while JNK2 knockout mice on a CDAA diet exhibited hepatic inflammation, steatosis, and fibrosis comparable to levels observed in normal mice [39]. Thus there may be differential roles for JNK1 and JNK2 in protection from liver injury, however these findings are not consistent across different models, and reduction of JNK2 expression does not consistently have deleterious effects.

Several study limitations deserve mention. The study was blinded and placebo-controlled only for the first 4 weeks, and study cohorts showed some imbalances despite the randomization. Both of these factors may have contributed to an overestimation of the effect of CC-930 on disease progression and exploratory efficacy parameters. In addition, the study had a small sample size per dose level, which may have limited assessment of the potential benefit/risk profile of CC-930. Also, the study was stopped early, due to elevated hepatic transaminase levels, as described above.

Several other agents have been evaluated in similar IPF populations, and two agents, pirfenidone and nintedanib, have recently been approved for use in the United States [40, 41]. The limited clinical efficacy of these drugs necessitates the development of additional compounds in order to effectively combat disease. The present study highlights the potential clinical utility of targeting JNK in settings of fibrotic lung remodeling via CC-930.

## Conclusion

In aggregate, we demonstrated that CC-930 attenuated phospho-c-Jun, MMP-7, and collagen deposition in a murine model of fibrotic airways remodeling. CC-930 attenuated phospho-c-Jun in human subjects, and was well tolerated during the initial 4-week, double-blind, ascending-dose phase, but elevated liver enzymes were observed in some patients without identifiable risk factors who were treated with higher doses for 8–12 weeks. In IPF patients treated with CC-930, a decline in FVC percent of predicted was observed after ≥6 months, although in the absence of a placebo control group beyond week 4 in this treatment period, no conclusions about the effect of CC-930 on pulmonary function can be made. Changes in FVC were significantly correlated with changes in plasma MMP-7, SP-D, and tenascin-C concentrations. Furthermore, the changes in plasma MMP-7 were associated with CC-930 dose and exposure. These findings demonstrate that it may be possible to use serum biomarkers to track disease progression in IPF patients as well as the potential clinical benefit of novel interventions.

## Additional file

10.1186/s40169-016-0117-2 Additional materials.

## Abbreviations

IPF: idiopathic pulmonary fibrosis
BALF: bronchoalveolar lavage fluid
MMP: matrix metalloproteinases
FVC: forced vital capacity
DL_CO_: diffusing capacity for carbon monoxide
SP-A: surfactant protein A
SP-D: surfactant protein D
KL-6: Krebs von den Lungen-6 antigen
MCP-1: monocyte chemotactic protein-1
TIMP-1: tissue inhibitor of metalloproteinase-1
PAI-1: plasminogen activator inhibitor-1
JNK: c-Jun N-terminal Kinase
TGF-β1: transforming growth factor β1
HDM: house dust mite
AE: adverse events
BID: bis in die (twice a day)
QD: quaque die (daily)
ECG: electrocardiogram
PAS: periodic acid-Schiff
α-SMA: α-smooth muscle actin
ALT: alanine aminotransferase
AST: aspartate aminotransferase
