# Supplementary material for: JNK inhibition reduces lung remodeling and pulmonary fibrotic systemic markers
Source: Clin Transl Med. 2016 Sep 2;5(1):36. doi: 10.1186/s40169-016-0117-2 (PMC5010551; doi:10.1186/s40169-016-0117-2)
Supplement: Supplementary file 1 — 10.1186/s40169-016-0117-2 Additional materials. [file 40169_2016_117_MOESM1_ESM.docx]

**Supplemental Material**

**JNK inhibition reduces lung remodeling and pulmonary fibrotic systemic markers**

Jos L. J. van der Velden,^1^ Ying Ye,^2^ James D. Nolin,^1^ Sidra M. Hoffman,^1^ David G. Chapman,^1^ Karolyn G. Lahue,^1^ Sarah Abdalla,^1^ Peng Chen,^3^ Yong Liu,^2^ Brydon Bennett,^4^ Nasreen Khalil,^5^ Donna Sutherland,^3^ William Smith,^3^ Gerald Horan,^2^ Mahmoud Assaf,^2^ Zebulun Horowitz,^3^ Rajesh Chopra,^2^ Randall M. Stevens,^3^ Maria Palmisano,^2^ Yvonne M. W. Janssen-Heininger,^1^ and Peter H. Schafer^2^

^1^Department of Pathology, University of Vermont, Burlington, Vermont, USA. ^2^Department of Translational Development, Celgene Corporation, Summit, New Jersey, USA. ^3^Clinical Research and Development, Celgene Corporation, Warren, New Jersey, USA. ^4^Department of Inflammation Research, Celgene Corporation, San Diego, California, USA. ^5^Division of Respiratory Medicine, University of British Columbia, Vancouver, British Columbia, Canada.

Address correspondence to: Peter H. Schafer, Department of Translational Development, Celgene Corporation, 86 Morris Avenue, Summit, NJ 07901, USA. Tel.: +1-908-673-9166. Fax: +1-908-673-2792. E-mail: [pschafer@celgene.com](mailto:pschafer@celgene.com).

**Supplemental Figure 1. House dust mite (HDM)-induced pro-fibrotic gene expression. Assessment of mRNA abundance of (A) α-SMA and (B) E-cadherin, and (C) surfactant protein D. mRNA abundance was normalized to β-actin.** Results are expressed as fold change compared with phosphate-buffered saline (PBS) exposed mice and reflect mean ± SEM from 2 independent experiments (PBS: *n* = 10; HDM alone: *n* = 10; HDM + vehicle control: *n* = 11; HDM + CC-930: *n* = 11).

**Supplemental Figure 2. Effect of CC-930 on house dust mite (HDM)-induced TGF-β1 production and airway hyperresponsiveness.** (**A**) Evaluation of total TGF-β1 protein in bronchoalveolar lavage fluid (BALF) measured by the enzyme-linked immunosorbent assay.
(**B**) Changes in respiratory mechanics were analyzed in phosphate-buffered saline (PBS) or HDM-challenged mice 72 hours after the last HDM exposure. Ascending doses of methacholine were administered to determine airway resistance (Rn). The response to methacholine at each dose was quantified as the average of the 3 peak measurements for airway resistance (Rn). Data shown represent means ± SEM from 2 independent experiments (PBS: *n* = 10; HDM alone: *n* = 10; HDM + vehicle control: *n* = 11; HDM + CC-930: *n* = 11). **P* < 0.05 (analysis of variance) versus PBS.

**Supplemental Figure 3.** CC-930 does not affect house dust mite (HDM)-induced allergic airway inflammation. (**A**) Lung histopathology in mice exposed to phosphate-buffered saline (PBS), HDM alone, HDM + vehicle, or HDM + CC-930. Lung sections were stained with hematoxylin and eosin (magnification: x200). Inflammatory cells profile in bronchoalveolar lavage fluid. (**B**) Total cells, (**C**) eosinophils, (**D**) Lymphocytes, and (**E**) macrophages were enumerated. Data shown represent means ± SEM from 2 independent experiments (PBS: *n* = 10; HDM alone: *n* = 10; HDM + vehicle control: *n* = 11; HDM + CC-930: *n* = 11). **P* < 0.05 (analysis of variance) versus PBS.

**Supplemental Figure 4.** CC-930 Reduces Ultraviolet Light-induced C-Jun Phosphorylation in Phase 1 Healthy Volunteer Study. (A) Representative Images of Phospho C-Jun Immuno-Staining by Treatment from Subject No. 15. Phospho c-Jun IHC images were scored by 3 independent evaluators (see phosphor-c-Jun IHC scores in inset within each image). (B) Scattered Plot of Individual Phospho c-Jun and Median Scores by CC-930 Treatment. Line across points denotes median value.

A

B

**Supplemental Figure 5. Idiopathic pulmonary fibrosis clinical study design.**

**Supplemental Figure 6. Idiopathic pulmonary fibrosis patient disposition.**

**Supplemental Figure 7. CC-930 pharmacokinetics in idiopathic pulmonary fibrosis patients.** Mean CC-930 plasma concentration-versus-time profile in 4 patients after a single 100-mg dose and multiple doses of 100 mg QD for 2 weeks are shown. (**A**) Mean CC-930 plasma concentration-versus-time profile at Week 0 and Week 2 (pharmacokinetic population) and (**B**) population pharmacokinetic model-predicted CC-930 area under the plasma drug concentration-versus-time curve during the dosing interval (AUC_0-τ_). ^a^Seven patients were randomized to CC-930 100 mg BID and 1 patient was randomized to placebo. A protocol amendment reduced the CC-930 dose to 100 mg QD effective immediately for patients still on study drug; 3 patients were affected by this dose reduction; of these, 3 patients had their dose reduced to 100 mg BID due to a protocol amendment.

**Supplemental Methods**

**Animals and reagents**

C57BL\6 mice were purchased from Jackson Laboratories (Jackson Laboratory, Bar Harbor, ME) and were maintained as a breeding colony under pathogen-free conditions. Mice were housed with a 12:12 hour light:dark cycle and allowed free access to standard laboratory chow and water. All experiments were conducted with age matched wild-type littermate controls. All animal studies were approved by the Institutional Animal Care and Use Committee (IACUC) at the University of Vermont. All chemicals utilized were purchased from Sigma-Aldrich (St Louis, MO) unless otherwise noted. Phospho-c-Jun antibody was obtained from Cell Signaling Technology (Danvers, MA); antibodies to α-SMA and actin were from Santa Cruz Biotechnology (Santa Cruz, CA); and antibodies to MMP-7 and MUC5B were from ABCAM (Cambridge, MA).

**HDM administration**

House dust mite (HDM) *Dermatophagoides pteronyssinus* extract (Greer Laboratories, Lenoir, NC) was resuspended in sterile PBS at a concentration of 1 µg (protein)/mL. The control group was sham sensitized with PBS. The experimental groups were sensitized with intranasal instillation of 50 µL of the HDM suspension once daily for 5 out of 7 days per week over 3 consecutive weeks (Figure 1A). During the HDM exposure, mice were treated with either vehicle (0.5% CMC/0.25% Tween 80) or CC-930 100 mg/kg BID (8 hours split between doses) orally in a volume of 5 mL/kg for the entire duration of the experiment. Mice were euthanized 72 hours after the final instillation of HDM.

**Collection and analyses of bronchoalveolar lavage and serum**

Lungs were lavaged with 1.0 mL of 1 x Dulbecco’s PBS. Total and differential cell counts were performed. Brieﬂy, cells were isolated by centrifugation and total cell counts were performed using the Advia 120 (Siemens, Malvern, PA) automated hematology analyzer. Cytospins were performed and stained using the Hema3 kit (Fisher Scientific, Kalamazoo, MI). Differential cell counts were performed on a minimum of 300 cells. Bronchoalveolar lavage fluid was centrifuged at 1200 x g for 5 minutes to remove cells and debris and was then snap-frozen in liquid nitrogen. Bronchoalveolar lavage fluid TGF-β1 was determined by ELISA kit (R&D Systems, Minneapolis, MN). Bronchoalveolar lavage fluid MMP-7 and MUC5B protein levels were determined by ELISA kits (Biocompare, San Francisco, CA). Following euthanization, blood was collected by heart puncture and immediately spun through a microtainer, and serum was separated. Analysis of serum MMP-7 was performed via ELISA methods according to the manufacturer’s instructions (Biocompare, San Francisco, CA).

**Homogenization of lung tissue and Western blotting**

Protein lysates were prepared by mincing lung tissue in cold lysis buffer immediately followed by homogenization as previously described (1). Lysates were incubated on ice for 30 minutes, followed by 30 minutes of centrifugation at 16,000 x g. A portion of the supernatant was saved for protein determination, before the addition of Laemmli sample buffer. Total protein was assessed by the Bio-Rad DC Protein Assay kit (Bio-Rad, Hercules, CA). Phospho-c-Jun, α-smooth muscle actin, and β-actin protein abundance was evaluated by Western blotting.

**Assessment of inflammation and mucus metaplasia**

Lungs were inflated to 25 cm of H_2_O and fixed with 4% paraformaldehyde in PBS followed by paraffin imbedding. Paraffin blocks were cut into 5-μm sections and mounted to slides. Tissue histopathology and inflammation were assessed by hematoxylin and eosin staining. In mice challenged with PBS and HDM, mucus metaplasia was assessed by periodic acid-Schiff (PAS) staining, and quantified by scoring airway PAS staining reactivity using a scale of 0 to 3 (0 representing no positive staining, 3 representing the highest intensity) by 2 independent, blinded observers. The cumulative score from each mouse was then averaged according to treatment group as described previously (1). MUC5AC expression was assessed via real-time PCR analysis.

**Assessment of airway mechanics and airway hyperresponsiveness**

Mice were anesthetized with intraperitoneal pentobarbital sodium (90 mg/kg), tracheotomized, and mechanically ventilated at 200 breaths/min with a tidal volume of 0.25 mL and positive end-expiratory pressure of 3 cm H_2_O (FlexiVent, SCIREQ, Montreal, QC, Canada). Airway mechanics were assessed by the forced oscillation technique in which respiratory impedance was partitioned using the constant phase model into the measure of Newtonian resistance (Rn) (2). Airway responsiveness was assessed during airway challenge with increasing doses of aerosolized methacholine (saline control, 12.5, 25, and 50 mg/mL), as previously described (3). The response to methacholine at each dose was quantified as the average of the 3 peak measurements.

**Assessment of HDM-induced collagen deposition**

Lung sections were stained with Masson’s trichrome reagent to stain collagen. Slides were scored using a scale of 0 to 3 (0 representing the least stain intensity, 3 representing the highest intensity) for airway-associated collagen deposition by 2 independent, blinded investigators. The cumulative score from each mouse was then averaged according to treatment group. Total lung collagen was measured in the upper right lobe of the lung after overnight digestion with 10 mg/mL pepsin in 0.5 M acetic acid using the Sircol Assay (Biocolor, Carrickfergus, UK) as directed by the manufacturer.

**Determination of hydroxyproline in lung tissue samples**

Hydroxyproline content was assessed in the right upper lobe of the lung as previously

described (4). Briefly, lung tissue samples were weighed and dried in an oven at 90°C overnight, and the weight was recorded again. Dry tissues were then boiled in 0.5 mL of 6 M HCl at 120°C overnight. After cooling down and adding 5 μL of phenolphthalein (1%), the samples were neutralized with NaOH 10 M and 6 M HCl. Black precipitate and brown color were removed by adding 100 μL of carbon, centrifugation, and filtration. Five microliters of standard or hydrolyzed samples were pipetted in triplicate onto a 96-well plate. Five microliter of citrate acetate buffer (5% citric acid, 7.2% sodium acetate, 3.4% sodium hydroxide, 1.2% glacial acetic acid, distilled water) and 100 μL of freshly prepared chloramine-T solution (14.1 mg chloramine-T, 0.1 mL n-propanol, 0.1 mL distilled water, 0.8 mL citrate acetate buffer) were added to each well. The samples were then incubated at room temperature for 20 minutes. After adding 100 μL of Ehrlich’s reagent (2.5 g of 4-(dimethylamino) benzaldehyde, 9.3 mL of n-propanol, and 3.9 mL of 70% perchloric acid), the wells were incubated for 20 minutes at 65°C. After cooling down, the samples were measured at 550 nm, a standard curve from 5 to 100 mg hydroxyproline in water was created. Hydroxyproline data were expressed as micrograms per right lung lobe.

**MMP-7 IHC**

MMP-7 staining was performed on lung sections after antigen retrieval by incubation of slides for 20 minutes in 0.01M sodium citrate pH 6.0 at 95ºC. Slides were then blocked with 2% normal goat serum for 30 minutes, followed by incubation with monoclonal rabbit antibody against MMP-7 (1:500 dilution; ABCAM) overnight at 4ºC. Biotinylated anti-rabbit IgG was then applied for 30 minutes at room temperature, followed by addition of the avidin-biotin- complex-alkaline phosphatase (Vectastain ABC-AP, Vector Laboratories, Burlingame, CA) for another 30 minutes at room temperature. After rinsing the sections in PBS, the substrate, Vector Red (Vector Laboratories), was added for 20 minutes. The Vector Red reacts with the bound alkaline phosphatase, producing an intense red color. Slides were counterstained with Mayerʼs hemotoxylin.

**RNA isolation and qPCR**

RNA was extracted using RNeasy columns (Qiagen, Inc., Valencia, CA) as directed by the manufacturer. Gene expression analysis was performed by reverse transcriptase-qPCR using the miScript Reverse Transcription kit and SYBR Green PCR kit (Bio-Rad, Hercules, CA). PCR data were analyzed by using the ∆∆Ct method of relative quantification. Primer sequences were taken from Genbank. All accession numbers are listed below.

| ***Gene*** | ***Accession*** |  | ***Sequences (5´→ 3´)*** |
| --- | --- | --- | --- |
| MMP7 | NM_010810.4 | forward | atcagtgggaacaggctcag |
|  |  | reverse | ttctgcaacatctggcactc |
| MUC5B | NM_028801.2 | forward | ctggccagtttgaaggagac |
|  |  | reverse | ttggttgtcactctgcttgc |
| Acta2 (α-SMA) | NM_007392.2 | forward | ctgacagaggcaccactgaa |
|  |  | reverse | catctccagagtccagcaca |
| Col1A1 | NM_007742.3 | forward | gagcggagagtactggatcg |
|  |  | reverse | gttcgggctgatgtaccagt |
| Col4a1 | NM_009931.2 | forward | aaagggagaaagaggcttgc |
|  |  | reverse | cctttgtaccgttgcatcct |
| Col5a1 | NM_015734.2 | forward | ggtccctgacacacctcagt |
|  |  | reverse | tgctcctcaggaacctctgt |
| Muc5ac | NM_010844.1 | forward | gctacacccaggttgagaagtg |
|  |  | reverse | tcctcactttccttggacttga |
| Ecadherin | NM_017474 | forward | gtctaccaaagtgacgctgaa |
|  |  | reverse | ggaggtgagtcaaggtgaga |
| Actin | NM_007393.3 | forward | ctgaatggcccaggtctga |
|  |  | reverse | ccctcccagggagaccaa |
| Sftpd (SPD) | NM_009160.2 | forward | atcagtacccaacacctgca |
|  |  | reverse | tctccctttggtccaggttc |

*Statistical analysis.* Data were evaluated using SPSS (version 21) by 1-way analysis of variance using the Tukey test to adjust for multiple comparisons. Results with *P* < 0.05 or smaller were considered statistically significant.

**References**

1. Alcorn JF, van der Velden J, Brown AL, McElhinney B, Irvin CG, Janssen-Heininger YMW. c-Jun N-terminal kinase 1 is required for the development of pulmonary fibrosis. *Am J Respir Cell Mol Biol.* 2009;40:422-432.
2. Tomioka S, Bates JH, Irvin CG. Airway and tissue mechanics in a murine model of asthma: alveolar capsule vs. forced oscillations. *J Appl Physiol.* 2002;93:263-270.
3. Riesenfeld E, Allen GB, Bates JH, et al. The temporal evolution of airways hyperresponsiveness and inflammation. *J Allergy Ther.* 2012;1:1-7.
4. Woessner, J.F., Jr. The determination of hydroxyproline in tissue and protein samples containing small proportions of this imino acid. Arch Biochem Biophys 1961; 93:440-447
